# A novel closure technique for a mucosal defect after endoscopic submucosal dissection: “closure on traction using a rotatable clip with a line loop”

**DOI:** 10.1055/a-2721-9439

**Published:** 2025-11-06

**Authors:** Yorinari Ochiai, Minoru Oda, Junji Tanaka, Yusuke Kawai, Hiroshi Yamato, Yugo Suzuki, Shu Hoteya

**Affiliations:** 1 13600 Department of Gastroenterology, Toranomon Hospital, Tokyo, Japan; 2 Okinaka Memorial Institute for Medical Research, Tokyo, Japan

Endoscopic submucosal dissection (ESD) is widely performed for early gastrointestinal tumors; however, delayed bleeding is a major complication, reported in 5–10% of cases 1 . Various closure methods after ESD may reduce delayed bleeding 2 3 .

We have developed and used a novel closure technique named “closure on traction using a rotatable clip with a line loop (CONTROLL)” to treat a mucosal defect after ESD ( Fig. 1 ), using only rotatable clips and a line loop completed in the lumen, without scope reinsertion.

**Fig. 1:**
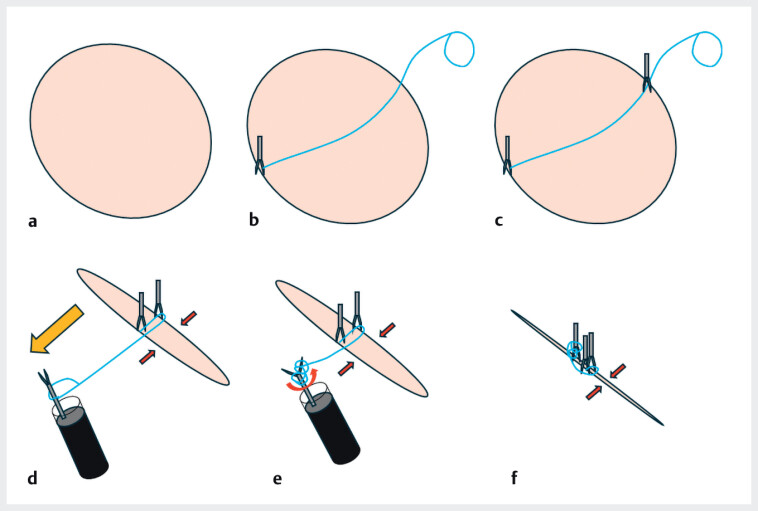
Schematic of the closure on traction using a rotatable clip with a line loop (CONTROLL) technique. **a** A post-endoscopic submucosal dissection ulcer defect. **b** First rotatable clip with a line loop placed at one side of the ulcer edge. **c** Second rotatable clip placed on the other side of the ulcer, including the line. **d** The third clip passed through the loop and moved to the first clip side for traction. **e** The opened third clip rotated to wrap the line around the clip. **f** By rotating the third clip, the line was shortened, resulting in a mucosa–mucosa traction, and the third clip was placed easily near the first and second clips.

A 71-year-old man underwent ESD for an early gastric cancer on the posterior side of the greater curvature of the antrum. Mucosal defect closure for an approximately 25-mm post-ESD ulcer was performed using CONTROLL. Before the procedure, a clip with a line with a 5–10-mm loop was set to a length approximately of 5-10 mm longer than the ulcer size ( Fig. 2 ). The first clip (SureClip; Micro-Tech Co., Ltd, Nanjing, China) with a line loop (polyester suture; Shirakawa Co., Ltd, Tokyo, Japan) was placed on one edge of the ulcer, including the muscular layer. A second clip (SureClip) grasping a line was deployed on the other side, including the muscular layer. A closed third clip (MANTIS Clip; Boston Scientific Corporation, Marlborough, MA, USA) was passed through the loop and moved to the side of the first clip. The third clip was opened and rotated to wrap a line around it. The edges of the ulcer simultaneously came close to the traction by shortening the line, and the third clip was placed near the first and second clips ( Fig. 3 and Video 1 ). Additional clips reinforced the defects. A follow-up endoscopy performed 3 days after the procedure showed no ulcer dehiscence ( Fig. 4 ).

**Fig. 2:**
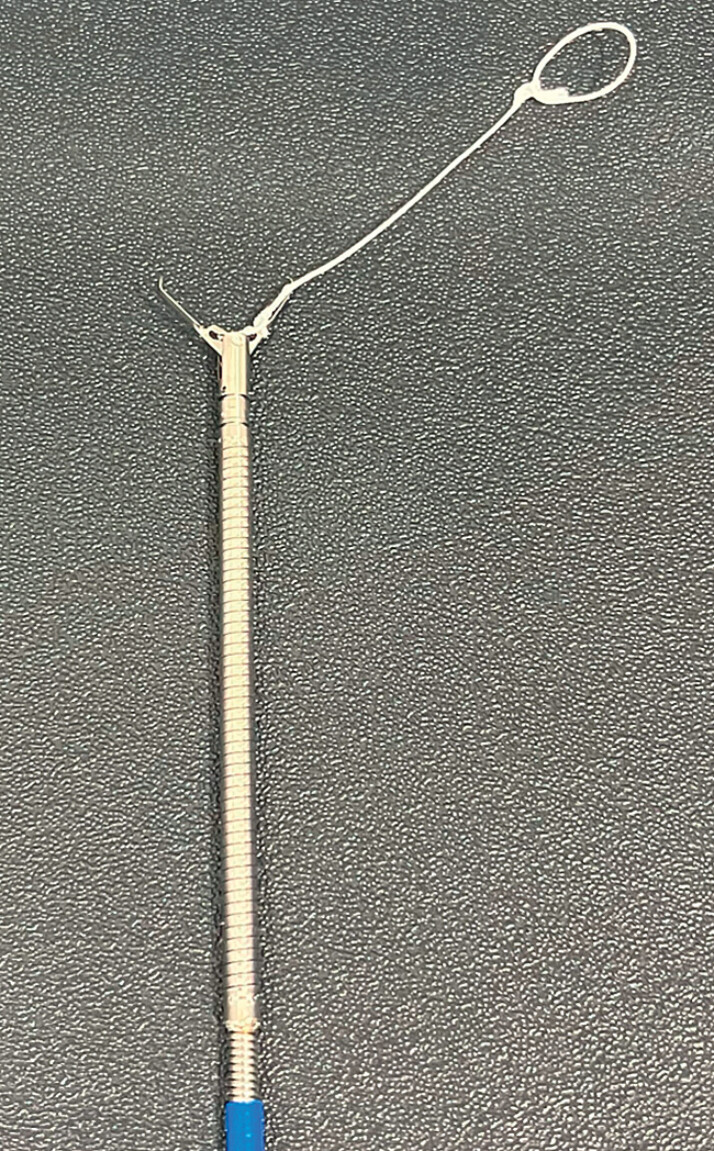
First clip with a line loop. Making a 5–10-mm loop and setting the length of the line as approximately 5-10 mm longer than the ulcer size. We used SureClip; (Micro-Tech Co., Ltd, Nanjing, China) and polyester suture (Shirakawa Co.,Ltd, Tokyo, Japan) in this case.

**Fig. 3:**
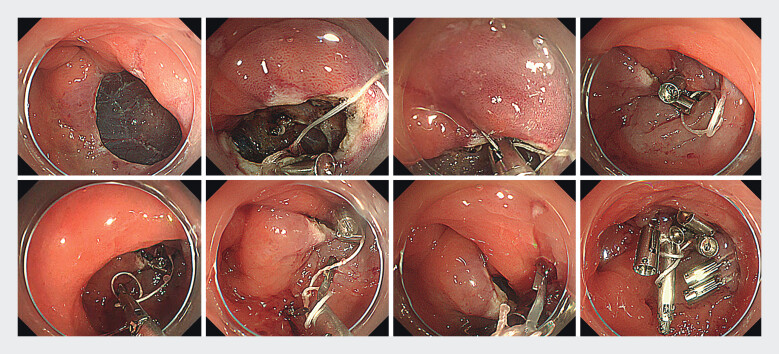
Endoscopic images of closure on traction using a rotatable clip with a line loop (CONTROLL) technique for endoscopic submucosal dissection ulcer defect.

A case of closure on traction using a rotatable clip with a line loop (CONTROLL) technique for a post-endoscopic submucosal dissection ulcer defect.Video 1

**Fig. 4:**
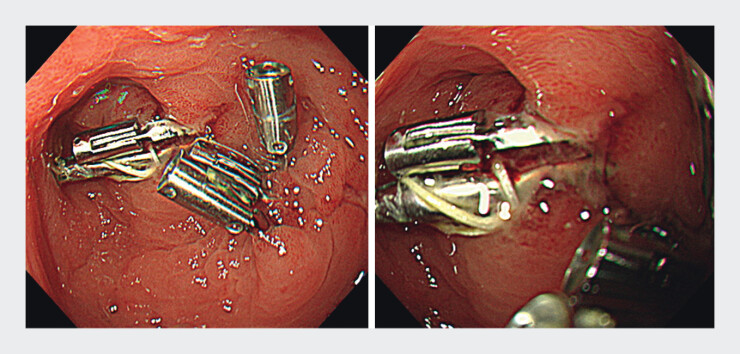
Endoscopic images 3 days after closure on traction using a rotatable clip with a line loop (CONTROLL) technique.

CONTROLL enabled firm closure. The clip–line and clip–clip connections become stronger by rotating the clip and shortening the line.

Endoscopy_UCTN_Code_TTT_1AQ_2AB

## Notes

Endoscopy E-Videos https://eref.thieme.de/e-videos
*E-Videos* is an open access online section of the journal *Endoscopy* , reporting on interesting cases and new techniques in gastroenterological endoscopy. All papers include a high-quality video and are published with a Creative Commons CC-BY license. Endoscopy E-Videos qualify for HINARI discounts and waivers and eligibility is automatically checked during the submission process. We grant 100% waivers to articles whose corresponding authors are based in Group A countries and 50% waivers to those who are based in Group B countries as classified by Research4Life (see: https://www.research4life.org/access/eligibility/ ). This section has its own submission website at https://mc.manuscriptcentral.com/e-videos .
